# Comparison of *Yersinia enterocolitica* DNA Methylation at Ambient and Host Temperatures

**DOI:** 10.3390/epigenomes7040030

**Published:** 2023-11-30

**Authors:** Dustin J. Van Hofwegen, Carolyn J. Hovde, Scott A. Minnich

**Affiliations:** Department of Animal Veterinary and Food Science, University of Idaho, Moscow, ID 83843, USA; djvanhofwegen@unwsp.edu (D.J.V.H.); cbohach@uidaho.edu (C.J.H.)

**Keywords:** *Yersinia*, temperature regulation, pathogenesis, DNA methylation, methylome

## Abstract

Pathogenic bacteria recognize environmental cues to vary gene expression for host adaptation. Moving from ambient to host temperature, *Yersinia enterocolitica* responds by immediately repressing flagella synthesis and inducing the virulence plasmid (pYV)-encoded type III secretion system. In contrast, shifting from host to ambient temperature requires 2.5 generations to restore motility, suggesting a link to the cell cycle. We hypothesized that differential DNA methylation contributes to temperature-regulated gene expression. We tested this hypothesis by comparing single-molecule real-time (SMRT) sequencing of *Y. enterocolitica* DNA from cells growing exponentially at 22 °C and 37 °C. The inter-pulse duration ratio rather than the traditional QV scoring was the kinetic metric to compare DNA from cells grown at each temperature. All 565 *Yen*I restriction sites were fully methylated at both temperatures. Among the 27,118 DNA adenine methylase (Dam) sites, 42 had differential methylation patterns, while 17 remained unmethylated regardless of the temperature. A subset of the differentially methylated Dam sites localized to promoter regions of predicted regulatory genes including LysR-type and PadR-like transcriptional regulators and a cyclic-di-GMP phosphodiesterase. The unmethylated Dam sites localized with a bias to the replication terminus, suggesting they were protected from Dam methylase. No cytosine methylation was detected at Dcm sites.

## 1. Introduction

The adaptation of facultative bacterial pathogens to their mammalian host environment requires significant global changes in gene regulation to establish infection. Host cues utilized for this transition vary among pathogens, but for many bacteria, the host temperature is a key environmental signal [1]. Temperature sensing is especially prominent in the pathogenic *Yersinia*. *Y. enterocolitica*, a Gram-negative enteropathogen, shows significant phenotypic changes between the narrow range of 30 °C and 37 °C (host temperature). Changes include a temperature-dependent requirement for calcium ions (2.5 mM at 37 °C) [2,3], modification of LPS acylation to circumvent Toll-like receptor (TLR) 4 stimulation [4,5], metabolic differences such as urease and acetoin production [6], and the reciprocal temperature-controlled regulation between two type-III secretion systems (TTSS). The latter includes the immediate repression of flagellum biosynthesis and concomitant induction of the pYV (virulence plasmid) TTSS at 37 °C [7,8]. The reciprocal temperature regulation of these two TTSS may be required because of the substrate reciprocity of their exported proteins [7]. The co-expression of flagella and the virulence TTSS at 37 °C would result in the injection of flagellin into host cells by the pYV-encoded TTSS. Flagellins are potent cytokine inducers of TLRs 5 and Ipaf, and their injection into host cells could effectively countermand the pYV-TTSS effectors, termed *Yersinia* outer proteins (Yops). The Yops collectively act to suppress the host innate immune system. Conversely, the flagellar TTSS exports Yops into the host extracellular milieu, rather than a direct injection into host cells, diluting their effect. Thus, the immediate cessation of flagellin expression and concomitant induction of the Yops may be essential during the initial stages of host infection.

In contrast to the *Y. enterocolitica* rapid response to the host temperature, acclimation to 25 °C after a temperature downshift (37 °C to 25 °C) is much slower. *Y. enterocolitica* adapted to 37 °C and downshifted to 25 °C, requires ~2.5 generations before flagellins (*fleABC*) are expressed [8]. The relative timing varies between four and 10 h, depending on the growth rate (rich vs. minimal medium), but the 2.5 generation requirement is consistent. This suggests restoration of a low temperature phenotype is cell-cycle dependent. A model of temperature-dependent differential DNA methylation could link temperature-regulated genes to the cell cycle if the expression of a key regulatory gene was sensitive to DNA methylation. This is because two generations of DNA replication are required for a DNA site to go from a fully methylated to an unmethylated state.

Bacterial DNA methylation occurs at adenine and cytosine bases providing epigenetic information in the form of N6-methyladenine (N6mA), N4-methylcytosine (N4mC), and 5-methylcytosine (5mC). DNA methylation is a mechanism to discriminate self from non-self (restriction sensitive), direct mismatch repair, excise non-methylated, i.e., non-self, strands of DNA [9], and activate transpositions [10]. Recent studies expand the role of bacterial DNA methylation to include regulation of cell cycle progression [11,12] and modulation of gene expression [13,14,15]. For example, DNA replication forks leave a wake of transient hemi-methylated sites on the newly synthesized DNA strand. Hemi-methylation of gene promoters can either activate or repress expression by promoting or inhibiting the binding of specific transcription factors. Transient hemi-methylation states associated with the passing of DNA replication forks account for the link between *Caulobacter crescentus* developmental gene regulation and its cell cycle [11] and the frequency of *E. coli Tn*10 transposition [16]. Thus, the modulation of DNA methylation provides a nondestructive and reversible means of DNA modification. This biphasic epigenetic switch is one mechanism organisms use to sense and adapt to their environment [17]. Recent reviews of bacterial methylation include Seong et al. and Gao et al. [18,19].

Temperature modulations of DNA supercoiling, histone-like protein DNA binding, and intrinsic DNA bends also contribute to *Y. enterocolitica* host adaptation. DNA supercoiling levels naturally respond to changes in temperature to control expression of flagella and virulence factors or can be artificially manipulated using gyrase inhibitors or novobiocin-resistant mutants [8]. Intrinsic DNA bends associated with poly-A and T tracts are sensitive to temperature and effectively melt at 37 °C. Changes in DNA structure affect the binding of histone-like proteins, promoter function, and methylation [20]. Coupling temperature-regulation to DNA methylation is also evident in *Yersinia*. The overproduction of DNA adenine methylase (Dam) in *Y. pseudotuberculosis* overrides the low temperature repression of Yop expression but not the low Ca^2+^ requirement for Yop secretion [21]. Dam methylates the N6 position of adenine in 5′-GATC-3′ sequences. In *Y. enterocolitica*, an overproduction of Dam alters motility and invasion and results in increased amounts of rough lipopolysaccharide (LPS) lacking O-antigen side chains [22]. Together, these results suggest that Dam sites may show variation in DNA methylation in different environmental conditions including temperature.

Until recently, high-throughput analyses of genomic epigenetic markers were limited to analyzing 5mC via bisulfite conversion followed by sequencing, such as Sanger, pyrosequencing, or whole genome amplification [23,24]. The limitation was significant since many studies indicate that bacterial DNA methylation centers on N6mA as the modified base [25,26,27]. With the advent of single-molecule real-time (SMRT) sequencing, a method for sequencing the methylome of bacteria to identify site modifications of adenine exists [28,29,30]. SMRT sequencing allows for the analysis of the native prepared DNA without the requirement of a whole genome amplification, therefore revealing the DNA, as the organism has modified it in vivo [31].

In this study, we tested the hypothesis that the *Y. enterocolitica* genome has temperature-dependent differences in DNA methylation. We compared the methylome of *Y. enterocolitica* DNA isolated from cells grown at 22 °C and 37 °C, providing the first systematic analysis of the epigenetic modifications in this important pathogen. We identified Dam sites with disparate methylation patterns dependent upon growth temperature and characterized them in relation to promoter regions or coding sequences.

## 2. Results

### 2.1. DNA Sequence Analysis Confirmed Four Genes Encoding Potential Methyltransferase Enzymes

**Figure 1:**
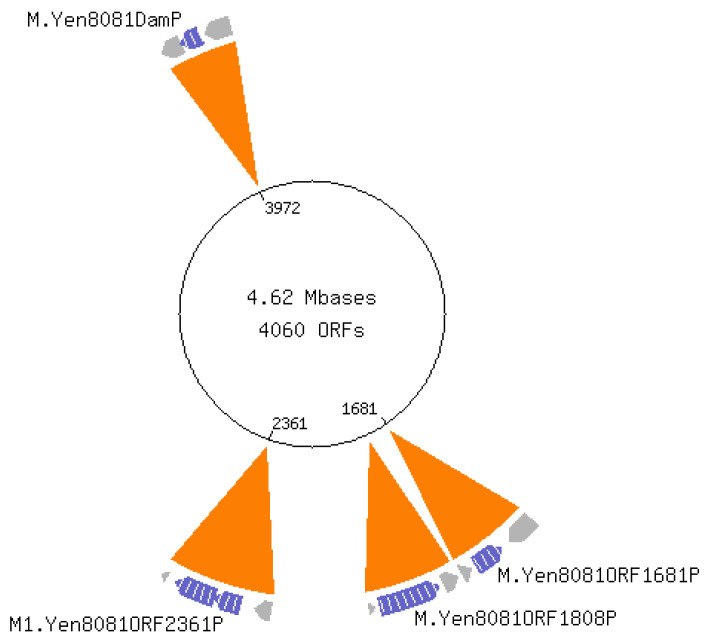
*Y. enterocolitica* sequence analysis identifies 4 methyltransferases.

The NEB REBASE database identifies four potential methyltransferase enzymes [32], which were confirmed by the genomic sequencing of *Y. enterocolitica* strain 8081 (Figure 1). The previously reported restriction modification (R-M) *yenI* system [33] was present at loci YE1808. The *yenI* operon encoded a restriction enzyme and a methyltransferase. The *Yen*I restriction enzyme is *Pst*I-like, recognizing the sequence 5′-CTGCAG-3′. Three ‘orphan’ methyltransferase enzymes, i.e., lacking a corresponding restriction enzyme, were also identified: YE3972 and YE2361 encoded DNA adenine methyltransferases (Dam) that recognize 5′-GATC-3′. Previous studies show that phenotypic changes following *dam* overexpression in *Yersinia* involve overexpression of loci YE3972 [22,34,35]. A third ‘orphan’ methylase, identified as YE2362, was a putative DNA cytosine methyltransferase (Dcm). The coding sequence for this putative Dcm overlapped with the 3′-end of the YE2361 Dam methylase.

The figure was generated by the REBASE program provided by New England Biolabs. YE0519 is a putative restriction endonuclease with unidentified motif specificity. YE1681 is a putative orphan methyltransferase with unidentified motif specificity; ‘orphan’ refers to the absence of a corresponding restriction endonuclease. YE1808 encodes the *Yen*I restriction modification system recognizing motif 5′CTGCAG-3′ previously described [33]. YE2361 is identified as a DNA adenine methyltransferase (Dam) recognizing 5′-GATC-3′. This ORF overlaps by 4 bp with the downstream ORF, YE2362, which, according to NCBI, encodes a DNA cytosine methyltransferase (Dcm). YE3972 is an orphan Dam having recognition specificity to 5′-GATC-3′ as reported previously Fälker et al. [22,34,35]

### 2.2. Inter-Pulse Duration Analysis Identified Methylated Nucleotides

**Figure 2:**
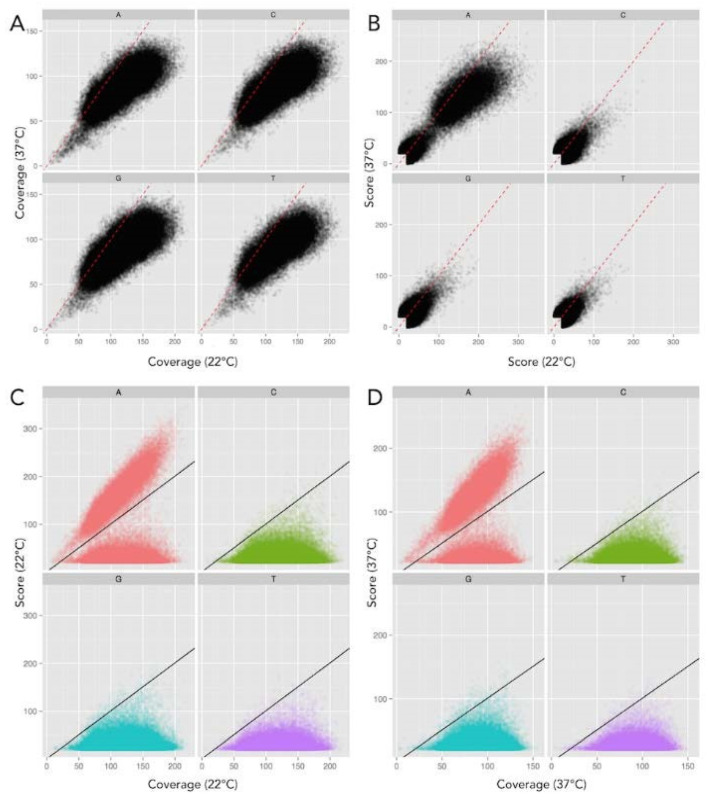
Base modification score to detect methylation is influenced by coverage bias. DNA methylation can be determined by an increased Quality Value (QV) score (−log10 (*p*-value)) compared to an unmodified in silico control (28). However, the scatter plot in panel (**A**) illustrates the coverage of the 37 °C sample (*x*-axis) is significantly higher than that of the 22 °C sample (*y*-axis), shown with a 1:1 correlation in the dotted red line. QV score has a strong dependence on coverage, increasing as coverage increases, thus Panel (**B**) reveals a similar trend when comparing scores as in Panel (**A**), with scores skewing toward the 37 °C sample. Therefore, this illustrates that QV scores alone provides a poor metric for comparison between each sample. (**C**,**D**) Scatter plot of sequencing coverage and QV score for all genomic positions at 22 °C and 37 °C respectively, again separated for each base. The threshold for detected base modification above background is indicated by the black line. Scatter plot distributions show significantly higher scores for many adenine (red) residues above the background, whereas no increased scores are evident for all other bases, thus indicating only adenine residues are methylated. Color code: Red = A, Green = C, T = Blue, and T = purple.

Our sequencing had increased read-depth coverage of DNA isolated from cells grown at 22 °C (222) compared to the sequencing read-depth coverage from cells grown at 37 °C (178). This difference is depicted in Figure 2A,B. The software package to analyze DNA genomic sequences by Pacific Biosciences includes a MEME-chip analysis to identify DNA base modifications. A benefit of SMRT sequencing is that two data types exist in which to determine these base modifications. A quality value (QV) score can be determined by calculating −10log (*p*-value) compared to a whole genome amplified (unmethylated) in silico control. However, QV scoring has a strong dependence on the sequence coverage (number of reads over a given position), so the reliability of this value increases as coverage increases.

**Figure 3:**
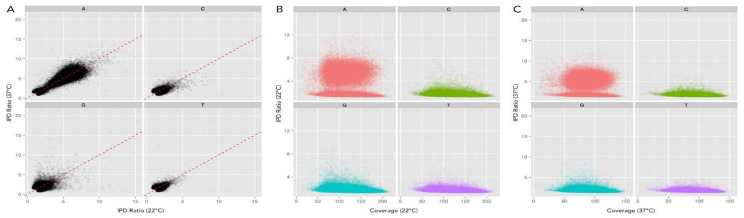
Inter-pulse duration (IPD) ratio provides an unbiased means of comparing samples with different coverage. (**A**) Scatter plot comparison of the IPD ratios for all residues from each sample, 22 °C on *x*-axis and 37 °C on *y*-axis, showing no bias toward either sample (1:1 correlation on dotted red line). This permits an unbiased comparison of each sample, indiscriminate of coverage, thus providing a good metric for comparison of each sample. (**B**,**C**) Scatter plot of sequencing coverage and IPD ratio for all genomic positions at 22 °C and 37 °C, respectively, separated for each base. Increased IPD ratio above baseline indicates DNA methylation. In each sample, many adenine (red) residues show an increased IPD ratio, revealing adenine methylation. Color code: Red = A, Green = C, T = Blue, and T = purple.

Base modifications can also be determined using the inter-pulse duration (IPD) ratio when sequenced samples differ in their coverage. Comparing the IPD ratio at each base across the genome using this method alleviated bias due to sequence coverage differences (Figure 3). Therefore, we used the IPD ratio, vs. QV scoring, as our kinetic metric to compare DNA prepared from cells grown at each temperature. 

Throughout, the following symbols depict DNA methylation states: ◼◼-denotes fully methylated sites; ◻◼-denotes hemi-methylated sites; and ◻◻-denotes unmethylated sites. In addition, patterns that varied with temperature are depicted as methylation conditions separated by an arrow indicating the shift from 22 °C to 37 °C, e.g., ◻◻➝◻◼.

### 2.3. Y. enterocolitica DNA Had No Evidence of Dcm (5mC) Methylation but Complete Methylation of All YenI Restriction Sites at Both 22 °C and 37 °C

There was no evidence of N4mC or 5mC base modifications (Figure 2 and Figure 3), despite the adequate sequence read-depth coverage. Therefore, we concluded that the Dcm methylase (YE2362) was not active in the LB-growth conditions at the two temperatures tested. In contrast, IPD analysis of all 565 *Yen*I (5′-CTGCAG-3′) restriction endonuclease sites showed that each adenine was methylated (N6mA) at both temperatures on both DNA strands (Table 1). This complete methylation was expected because these sequences are targets for DNA restriction. These sites are present throughout the genome so the identification of adenine methylation at these sites served as an internal control for identifying N6mA at non-*Yen*I sites. The genes and their chromosome positions are depicted in Figure 1.

### 2.4. Y. enterocolitica DNA Had Different Dam Methylation Patterns at 22 °C and 37 °C

Genomic DNA sequence analysis identified 27,118 5′-GATC-3′ Dam methylase sites in the *Y. enterocolitica* genome. The chromosome contained 26,664 sites and the pYV (virulence plasmid) contained 454 sites. IPD analysis showed that the majority of these Dam sites were fully methylated on both DNA strands at both temperatures analyzed (Table 1). Importantly, a subset of 42 Dam sites had different temperature-dependent methylation patterns (Table 2). In addition to these 42 sites, 17 Dam sites remained unmethylated at both temperatures (◻◻➝◻◻, Table 3). Temperature-dependent Dam methylation patterns were found throughout the genome in both regulatory and gene open reading frame sequences. The genes identified were classified as coding for putative regulators, ribosomal RNAs, membrane-associated proteins, metabolic proteins, virulence proteins, or for hypothetical proteins of no known function. We found no evidence of cytosine methylation in DNA from cells grown in these experimental conditions. Finally, all *Yen*I restriction endonuclease sites were fully methylated on both DNA strands at both temperatures. The specifics of the differential methylation are in Table 2 and Table 3 and outlined in the following paragraphs.

Two Dam sites were unmethylated at 22 °C and methylated at 37 °C (◻◻➝◼◼), localized in the 5′-regulatory regions of YE0914 and YE4070 at 36 and 100 bp, respectively, 5′ from the predicted AUG start codons (Table 2). YE0914 is identified by NCBI as a hypothetical protein, but EMBL’s Pfam sequence analysis identified it as a LysR-type transcription regulator (LTTR) [36]. LTTRs contain a helix-turn-helix DNA-binding domain and are among the most abundant type of transcriptional regulators within prokaryotes [37,38]. YE4070 codes for a putative outer membrane oligogalacturonate-specific porin, KdgM.

Four Dam sites were fully methylated at 22 °C and unmethylated at 37 °C (◼◼➝◻◻). These included the YE0335 (*hemN*) gene coding for coproporphyrinogen III oxidase with the Dam site 75 bp 5′ from the start codon. Two (◼◼➝◻◻) sites were within ribosomal RNA genes, both 16s rRNA and 23s rRNA, and one was within the coding region of YE1322, a putative RTX-family protein, associated with type I secretion system pore-forming toxins. YE1322, with a predicted open reading frame of 6333 bp, contains 17 additional Dam sites, four of which had full methylation at 22 °C and hemi-methylation at 37 °C (◼◼➝◼◻).

Of the seven Dam sites unmethylated at 22 °C and hemi-methylated at 37 °C (◻◻➝◻◼), five were in probable regulatory regions and two were within gene coding regions. Of the five Dam sites in intergenic regulatory regions, three were in genes identified as possible regulatory proteins. The first regulatory gene identified with this category was YE1259, with a Dam site 51 bp 5′ from the AUG start codon. YE1259 was identified as a PadR-like transcriptional regulator. The second regulatory gene in this category was YE3423, with a Dam site 85 bp 5′ from the AUG start codon. YE3423 codes for an ArsR-family transcriptional regulator. Of note, this Dam sites is positioned between two divergently transcribed genes, placing it 66 bp 5′ from the AUG start codon of YE3424. YE3424 codes for a putative zinc metallopeptidase. A BLAST search of the YE3424 predicted amino acid sequence had 70% identity and 83% similarity to enterohemorrhagic *E. coli* (EHEC) metallopeptidase, SprT, a type III secretion system effector. The third regulator gene in this category of Dam methylation (◻◻➝◻◼) was YE0316 with a Dam site 206 bp 5′ from the AUG start codon. YE0316 codes for putative DNA-binding protein with 53% similarity to sigma-70 (*fecI*). Of note, this Dam sites is also positioned 172 bp 5′ from the AUG start codon of divergently transcribed YE0315. YE0315 codes for a membrane transport protein with high similarity to *Salmonella typhimurium* TonB. Two additional genes showed this pattern of methylation (◻◻➝◻◼): YE0981, encoding a hypothetical protein with a Dam site 74 bp 5′ from the start codon; and YE1098 with the Dam site 51 bp 5′ from the start codon. This gene encodes GutA, also referred to as SrlA, a glucitol/sorbitol-specific IIC2 component, a subunit of the phoshotransferase system [39]. 

Of the 24 Dam sites fully methylated at 22 °C and hemi-methylated at 37 °C (◼◼➝◼◻), 22 were located within structural gene coding sequences (Table 2). Of the two sites in regulatory regions, one ◼◼➝◼◻ Dam site is positioned 288 bp 5′ from the AUG start codon of YE2225, a predicted cyclic-di-GMP phosphodiesterase with a conserved EAL domain. This enzyme inactivates cyclic-di-GMP, a common bacterial secondary messenger [40]. Interestingly, within the promoter of YE2225, three additional Dam sites had differential methylation patterns. The three Dam sites most proximal to the coding region, 38, 55, and 73 bp 5′ from the AUG start codon, remained unmethylated at each temperature. It is noteworthy that this regulatory region, from 38 to 288 5′ from the start AUG codon, contains four Dam sites that show atypical methylation patterns (Table 2 and Table 3). Statistically, only one Dam site was predicted over a span of 256 bp. The second Dam site in a potential regulatory region is on the virulence plasmid, 86 bp from the 5′ start of YEP0064, a putative pseudogene. This pattern of methylation (◼◼➝◼◻) was also prominent in rRNA genes. We identified five Dam sites in 16S and 23S rRNA genes, which were position-specific at the 3′-ends. This included four of the seven 16S ribosomal RNA genes (YEr007, YEr010, YEr018, and YEr022) with this conserved pattern of atypical methylation (Table 2 and Table 3).

Seventeen Dam sites were identified to be unmethylated at each temperature (◻◻➝◻◻). Ten were located in probable regulatory regions (Table 3). As described above, three unmethylated Dam sites were located 5′ from the start of YE2225, the putative cyclic-di-GMP phosphodiesterases. Two were at 131- and 144 bp 5′ from the start codon of YE0983, a hypothetical protein. The remaining unmethylated Dam sites were 5′ from the AUG start codon of an oligogalacturonate lyase, a bifunctional transaminase, a putative transporter, a putative glycosyl transferase, and YE2151, a TetR-like transcriptional regulator similar to NemR. (Table 3).

## 3. Discussion

The most significant finding of this work identified two subsets of Dam methylation sites with temperature-dependent Dam methylation patterns or Dam sites that remained unmethylated regardless of the temperature. To our knowledge, this is the first systematic analysis of temperature-dependent DNA methylation of a facultative bacterial pathogen. Importantly, the IPD ratio, rather than the traditional QV scoring, was the kinetic metric used to compare DNA so that the DNA sequence coverage differences between cells grown at different temperatures were not an issue. Both categories of Dam sites were localized either within regulatory regions (5′ from the predicted AUG start site) or within gene open reading frames. Among the genes with differential methylation were those encoding regulatory, virulence, metabolic, and membrane-associated proteins and both 23s and 16s rRNA genes. Importantly, we found no evidence of Dcm methylation at predicted Dcm sites (5′-CCWGG-3′) on the chromosome nor on the pYV. To determine if the temperature-dependent methylation patterns identified correlated with gene expression, we capitalized on the *Y. enterocolitica* comprehensive transcriptome analyzes conducted by Bent et al. [41]. This study is a comprehensive RNA-seq analysis of *Y. enterocolitica* 8081v transcripts prepared from cultures grown at 25 °C in LB broth and from 37 °C cultures grown in (i) conditioned RPMI, (ii) in surface contact with mouse macrophages, or (iii) internalized by mouse macrophages. 

The LTTR at loci YE0914 has a Dam site in the predicted regulatory region that is unmethylated at 22 °C and fully methylated at 37 °C (◻◻➝◼◼), exemplifying a gene that would require two rounds of DNA replication to restore the unmethylated state after a temperature downshift. This gene is highly conserved among the enteric bacteria with 88% identity to an LTTR in *Serratia marscesens* and 76% identity to an LTTR in *Shigella*. To our knowledge, neither YE0914 nor its enteric homologs have been characterized. LTTRs are among the most abundant types of transcriptional regulators present in bacteria and control a diverse subset of genes, including motility, metabolism, and virulence [38]. The *E. coli* LTTR, OxyR, activates one of its many targets, the phage Mu *mom* gene, only when three Dam sites upstream of the promoter are methylated [42]. Therefore, there is precedence for LTTR expression correlating with promoter methylation. Transcription of YE0914 in the RNA-seq transcriptomic analysis by Bent et al. [41] does not show temperature-dependent regulation between *Y. enterocolitica* cells grown in LB at 26 °C compared to conditioned RPMI at 37 °C. However, the *p*-value in this study for YE0914 at these two temperatures is very high (0.65–0.93), suggesting significant heterogeneity in expression within the sampled populations. Heterogenic expression is also indicative of epigenetic mechanisms whereby bacteria modify gene expression to “bet hedge” within otherwise clonal populations [43]. 

Among the regulatory genes identified with unmethylated Dam site(s) (◻◻➝◻◻) upstream of the translational start was the TetR-like transcriptional regulator YE2151. This gene has a predicted 64% amino acid similarity (52% identity) to *E. coli* NemR [44]. NemR activates several stress-response genes required for survival when cells are exposed to reactive oxygen species such as hypochlorus acid [45]. The transcriptomic analysis by Bent et al. [41] shows a significantly increased expression of YE2151 at elevated temperature. However, based on our methylation analysis, methylation did not appear to play a role in regulation since the unmethylated state of the Dam site identified did not change with temperature. It is noteworthy that this stress-response gene is localized near the terminus of DNA replication. Genes expressed during stress or the stationary phase are positionally biased to be near the terminus and show a requirement for reduced supercoiling for expression [46,47]. Previous studies by our laboratory have likewise demonstrated that temperature regulation in *Y. enterocolitica* correlates to changes in DNA supercoiling. DNA methylation can affect histone-like protein binding which in turn dictates regional DNA domain confirmation. As such, a lack of methylation at the chromosome terminus may influence the gene expression indirectly through Dam site protection.

Also showing differential regulation in its upstream regulatory region was the regulatory gene, YE1259 (◻◻➝◼◻), coding for a PadR-like transcriptional regulator. PadR family transcriptional regulators are involved in *Vibrio* virulence. For example, AphA is involved in the regulation of motility and virulence in *Vibrio parahaemolyticus* [48]. In *V. parahaemolyticus,* both an LTTR (AphB) and AphA co-regulate acetoin production and motility [49,50]. Notably, these phenotypes are temperature regulated in *Y. enterocolitica,* where acetoin and flagella are only produced at a low temperature [51]. The transcriptomic analysis by Bent et al. [41], however, reveals little variation in the temperature expression of YE1259. YE1098, coding for GutA, also showed the same pattern of methylation as YE1259 (◻◻➝◼◻). Van der Woude et al. [52] reported that the Dam site 44 bp from the *E. coli gutABD* transcription start site is unmethylated. GutR and CRP compete for binding at this Dam site, and GutR binding prevents methylation. However, changing the Dam site in *E. coli* did not alter the expression, suggesting that methylation does not play a direct role in *gutABD* regulation. In *Y. enterocolitica,* we noted the presence of a putative CRP-binding site overlapping this Dam site, suggesting similar regulation. The fact that we saw a transition in the methylation pattern with growth temperature suggests that the methylation of this operon may be sensitive to other environmental conditions. Indeed, Bent et al. [41] show significant activation of *gutA* at 37 °C.

The most extensive atypical Dam methylation was seen in YE2225. Over a span of 288 bp upstream of the translational start site are four Dam sites, three of which remained unmethylated at both temperatures and one, the most distal from the AUG start site, went from fully methylated at 25 °C to hemi-methylated at 37 °C (◼◼➝◼◻). YE2225 encodes a putative cyclic-di-GMP phosphodiesterase (PDE) with a conserved EAL domain required for hydrolysis of the common bacterial signaling messenger di-c-GMP [40]. A transcriptomic analysis by Bent et al. [41] shows that this gene undergoes a steady upregulation when exposed to mouse macrophages grown in RPMI at 37 °C, reaching a ~5.5-fold peak increase at 60 min. Cyclic-di-GMP plays an essential role during environmental transitions of bacteria. Phenotypes governed by cyclic-di-GMP include virulence gene expression and the transition from the motile to sessile states during biofilm formation [53]. For the related *Y. pestis*, cyclic-di-GMP regulates exopolysaccharide production during biofilm formation in the flea vector. Hydrolysis of cyclic-di-GMP is essential in the transition from the flea ambient temperature to the mammalian host temperature. Bobrov et al. (53) show that mutational inactivation of HmsP, the *Y. pestis* cyclic-di-GMP phosphodiesterase, results in a 1000-fold reduction in virulence. Our results suggest that methylation in concert with a higher temperature may alter cyclic-di-GMP levels essential for host infection. Interestingly, in *V. cholera*, both AphA and AphB repress the activation of the EAL-containing *acgA*. AcgA further shows effects on motility, virulence, and biofilm formation [50]. 

YE0316 (◻◻➝◼◻) is a homolog of FecI. FecI is a sigma factor which regulates the expression of the ferric di-citrate uptake system clustered chromosomally in the iron regulatory region of *E. coli* [54]. The *fecI* gene is temperature-regulated in *E. coli*, with a higher expression at 37 °C, and controlled by the histone-like nucleoid structuring (H-NS) protein [55]. This temperature regulation is true for YE0316; expression is increased over eight-fold at 120 min following 37 °C exposure [41]. Notably, H-NS binding is affected by DNA methylation [56]. 

Several locations of disparate methylation patterns were within or near ribosomal RNA elements. Of the seven 16S rRNA genes present on the chromosome, four showed temperature variation in methylation. Similarly, of the seven 23S rRNA elements, five showed temperature variation in methylation. The positions for each of these Dam sites were localized to the 3′ end of the coding region. Furthermore, the ribosomal RNAs showing this methylation pattern were localized near the origin of replication. The genomic position does effect the expression levels [46,57].

Finally, we identified 17 Dam sites which remain unmethylated. This number is consistent with unmethylated sites determined for uropathogenic *E. coli* and *C. crescentus* [29,52,58,59]. This suggests that these sites are permanently protected by either binding by proteins, local DNA domain structure, or both. It is noteworthy that 14 of these 17 unmethylated sites are clustered in 10 genes positioned near the origin (five sites) or terminus of DNA replication (12 sites).

The lack of cytosine methylation detected in our analysis is consistent with the transcriptome analysis of Bent et al. [41]. Our interpretation of their data indicates either no or minimal expression of YE2361 (*dam)* and YE2362 (*dcm*) at all growth and temperature conditions monitored in their experiments. Conversely, *yenI* (YE1808) and *dam* (YE3972) are expressed constitutively with little temperature variation. 

An overproduction of Dam affects the motility and invasion abilities of *Y. enterocolitica*, implying that temperature-dependent phenotypic differences may be regulated by temperature-responsive differential methylation [13]. In this study, we showed that there is a subset of Dam sites displaying temperature-dependent methylation patterns. Of the Dam sites in regulatory regions, some patterns correlate with temperature regulation when compared to the RNA-seq data of Bent et al. [41]. Surprisingly, we did not see differences in the methylation of regulatory regions of key genes involved in TTSS temperature regulation, suggesting that if methylation is a component of motility and Yop expression, it is indirect. However, the mechanism underlying this regulation by Dam remains unclear, and the genes identified here provide targets for further investigation.

## 4. Materials and Methods

### 4.1. Bacterial Strains, Culture Media, and DNA Techniques

*Y. enterocolitica* strain 8081v (R^−^M^+^) [31] was grown to late exponential phase in Luria-Bertani broth at either 22 °C or 37 °C. The presence of the virulence plasmid (pYV) was verified both by PCR and plating on Congo Red LB plates in calcium-chelating conditions (CR-MOX). Genomic DNA was isolated using Sigma (St. Louis, MI, USA) GenElute Bacterial Genomic DNA Kit, according to the manufacturer’s protocol.

### 4.2. SMRT Sequencing

SMRT sequencing analyzes the rate at which nucleotides are incorporated on the complimentary replicating strand and compares these kinetics to an in silico control. The resulting inter-pulse duration (IPD ratio) provides a kinetic profile that identifies modified bases [60]. Libraries of *Y. enterocolitica* 8081v cultures grown at each temperature (22 °C and 37 °C) were prepared for SMRT sequencing via circular consensus sequencing using a library construction protocol described previously [61,62,63]. Briefly, native chromosomal and plasmid DNA preparations were sheared to an average size of 500 bp via adaptive focus acoustics (Corvaris, Woburn, MA, USA), and end-repaired, and A-tailed and hairpin adapters with a single T-overhang were ligated. Primers were annealed and sequenced on the Pacific Biosciences RS instrument using C2 chemistry. These libraries were sequenced to a mean coverage depth of 228 (22 °C) and 178 (37 °C) across the chromosome, and 149 and 68, respectively, across the virulence plasmid; average read length was 2656 and 2682, respectively. Reads were mapped to the reference genome (RefSeq NC_008791 and NC_008800 for the chromosome and virulence plasmid, respectively) using BLASR [62]. Base modification and motif detection were performed using the Modification and Motif Detection protocol in the software program SMRTPipe v.1.3.3. Positions with coverage of >25 and score (QV) of >40 were considered modified. The score (QV) equals −10*log (*p*-value), where the *p*-value was determined from a t-test between the sample and the in silico control derived from whole genome amplified (WGA, i.e., unmethylated) samples. Due to the lower read depth coverage on the virulence plasmid, sites of adenine methylation with coverage >10 and an IPD ratio above three were considered modified, and this was further confirmed because each point of modification was located at known methylation sites.

## 5. Conclusions

Organisms sense and respond to their environment, in part, by epigenetic variation mediated by DNA methylation. Pathogenic bacteria vary gene expression to allow survival and activate virulence systems in response to host temperature. *Yersinia enterocolitica*, a facultative intracellular pathogen, respond by immediately repressing flagella synthesis and inducing the virulence plasmid-encoded type III secretion system. In this work, we examined the locations of DNA methylation throughout the *Y. enterocolitica* genome. While most methylation target sites were fully methylated, we identified sites with disparate temperature-dependent methylation. Several of these sites were within promoter regions of predicted regulatory genes. Differences in DNA methylation in promoter sequences are often responsible for variations in transcription. Identification of these differences in methylation provide likely candidates for regulators responsible for temperature-dependent phenotypes. 

## Figures and Tables

**Table 1:** Comparison of *Yen*I, Dam, and Dcm methylation sites at 22 °C and 37 °C.

	Total # of Motifs	22 °C		37 °C	
		# of Atypical Methylation Detected	# of Fully Methylation Detected	# of Atypical Methylation Detected	# of Fully Methylation Detected
5′-GATC-3′ (Dam)					
Chromosome	26,664	28	26,636	50	26,614
pYV Plasmid	454	7	447	7	447
5′-CTGCAG-3′ (*Yen*I)					
Chromosome	554	0	554	0	554
pYV Plasmid	11	0	11	0	11
5′-CCWGG-3′ (Dcm)					
Chromosome	7237	0	0	0	0
pYV Plasmid	88	0	0	0	0

Methylated bases are underlined.

**Table 2:** Dam sites showing temperature-dependent methylation differences at 22 °C and 37 °C.

Position	22 °C ^a^	37°C ^a^	Name	Location ^b^	Cat. ^c^	Description
20357	◼◼	◻◻	YEr002	ORF	rR	23S rRNA
315457	◼◼	◼◻	YEr007	ORF, −160 from 3′ end	rR	16S rRNA
315631	◼◻	◼◻		+16 from 3′ end of 16S rRNA		
356036	◼◼	◼◻	YEr010	ORF, −158 from 3′ end	rR	16S rRNA
356210	◼◼	◼◻		+16 from 3′ end of 16S rRNA		
373662	◻◻	◼◻	YE0315	−172 from TSS	M	putative membrane transport proteins, similar to Salmonella TonB
			YE0316	−206 from TSS	MT	putative DNA-binding protein
399373	◼◼	◻◻	YE0335	−75 from TSS	MT	HemN, coproporphyrinogen III oxidase
623091	◻◻	◼◻	YE0546	ORF	M	putative glycosyltransferase
888993	◻◻	◼◻	YE0762	ORF	MT	CysD, sulfate adenylyltransferase subunit 2
1042835	◻◻	◼◼	YE0914	−36 from TSS	R	putative LysR-type transcriptional regulator
1104202	◻◻	◼◻	YE0981	−74 from TSS	H	hypothetical protein
1111932	◼◼	◼◻	YE0990	ORF	MT	AYP/GTP-binding protein
1113753	◼◼	◼◻	YE0992	ORF	H	hypothetical protein
1228189	◻◻	◼◻	YE1098	−182 from TSS	MT	GutA, pts system, glucitol/sorbitol-specific iic2 component
1403906	◻◻	◼◻	YE1259	−51 from TSS	R	putative PadR-like family transcriptional regulator
1468949	◼◼	◼◻	YE1322	ORF	V	putative RTX-family protein
1469615	◼◼	◼◻				
1471616	◼◼	◻◻				
1471736	◼◼	◼◻				
1471902	◼◼	◼◻				
1879399	◼◼	◼◻	YE1683	ORF	R	putative prophage encoded two-component system response regulator
1881251	◼◼	◼◻	YE1684	ORF	R	putative prophage encoded two-component system histidine kinase
2354334	◼◼	◼◻	YE2154	ORF. near 5′	MT	Rnt, ribonuclease T
2436018	◼◼	◼◻	YE2225	−288 from TSS	R	putative cyclic-di-GMP phosphodiesterase
2598581	◼◼	◼◻	YE2407	ORF	V	putative hemolysin
2602469	◼◼	◼◻	YE2409	+30 from 3′ end of mviN	MT	MviN, putative membrane-associated protein
2852693	◼◼	◼◻	YE2635	ORF		putative metallo-beta-lactamase superfamily protein
3352496	◼◼	◼◻	YE3082	ORF	MT	RfbX, putative O-antigen transporter
3548081	◼◼	◼◻	YEr017	ORF	rR	23S rRNA
3549372	◼◻	◻◻				
3550740	◼◼	◼◻	YEr018	+17 from 3′ end of 16S rRNA	rR	16S rRNA
3655022	◼◼	◼◻	YE3343	ORF. near 3′	MT	OutL, general secretion pathway protein L
3739389	◻◻	◼◻	YE3423	−85 from TSS	R	ArsR-family transcriptional regulator
			YE3424	−66 from TSS	M	putative zinc metallopeptidases
4243325	◼◼	◻◻	YEr022	+17 from 3′ end of 16S rRNA	rR	16S rRNA
4441739	◻◻	◼◼	YE4070	−100 from TSS	MT	putative oligogalacturonate-specific porin protein
2443	◼◼	◼◻	YEP0004	ORF	V	YopQ, virulence plasmid protein
2654	◼◻	◼◻		ORF		
15967	◼◻	◼◼	YEP0018	ORF	MT	LcrD, low calcium response locus membrane protein d
21305	◼◻	◼◻	YEP0026	ORF	V	YscP, putative type III secretion protein
23409	◼◻	◼◼	YEP0029	ORF	V	YscS, putative type III secretion protein
24586	◼◼	◼◻	YEP0031	ORF	V	YscU, putative type III secretion protein
25258	◼◻	◼◼		ORF		
45942	◼◼	◼◻	YEP0064	−86 from TSS	H	putative pseudogene
48481	◼◻	◼◼	YEP0066	−158 from TSS	M	putative YadA invasin
66135	◼◼	◼◻	YEP0096	ORF	MT	putative plasmid copy control protein

^a^ ◼◼, fully methylated; ◼◻, hemi-methylated; ◻◻, unmethylated; ^b^ ORF, open reading frame; TSS, translational start site. ^c^ H, hypothetical; R, regulator; rR, ribosomal RNA; M, membrane-associated; MT, metabolic; V, virulence.

**Table 3:** Dam sites unmethylated at both 22 °C and 37 °C.

Position	22°C ^a^	37°C ^a^	Name	Location ^b^	Cat. ^c^	Description
316977	◻◻	◻◻	YEr008	ORF	rR	23s rRNA
357556	◻◻	◻◻	YEr011	ORF	rR	23s rRNA
1105938	◻◻	◻◻	YE0983	−131 from TSS	H	hypothetical protein
1105951	◻◻	◻◻		−144 from TSS		
1998525	◻◻	◻◻	YE1808	ORF	MT	*Yen*I, methyltransferase-endonuclease
1998670	◻◻	◻◻				
2057696	◻◻	◻◻	YE1876	−106 from TSS	MT	Ogl, oligogalacturonate lyase
2351648	◻◻	◻◻	YE2151	−23 from TSS	R	transcriptional repressor NemR
2435768	◻◻	◻◻	YE2225	−38 from TSS	R	putative cyclic-di-GMP phosphodiesterase.
2435785	◻◻	◻◻		−55 from TSS		
2435803	◻◻	◻◻		−73 from TSS		
2656973	◻◻	◻◻	YE2469	−369 from TSS	MT	ArgM, bifunctional succinylornithine transaminase/acetylornithine transaminase
2857854	◻◻	◻◻	YE2639	−318 from TSS	M	putative transporter protein
3351041	◻◻	◻◻	YE3081	ORF	MT	WbcD, putative 6-deoxy-D-Gul transferase
			YE3080	−191 from TSS	MT	WbcE, putative glycosyl transferases
4241957	◻◻	◻◻	YEr021	ORF	rR	23s rRNA
4243004	◻◻	◻◻	YEt079	ORF		tRNA-Ala
65753	◻◻	◻◻	YEP0094	ORF	MT	IS541 transposase
			YEP0095	−240 from TSS	MT	putative plasmid copy number

^a^ ◻◻, unmethylated; ^b^ ORF, open reading frame; TSS, translational start site. ^c^ H, hypothetical; R, regulator; rR, ribosomal RNA; M, membrane-associated; MT, metabolic.

## Data Availability

All methylation/base modification data are available at figshare: https://dx.doi.org/10.6084/m9.figshare.3493247 accessed on 20 July 2016. and https://dx.doi.org/10.6084/m9.figshare.3493310 accessed on 20 July 2016.

## Abbreviations

SMRT—single-molecule real-time; IPD—interpulse duration; QV—quality value; PDE—phosphodiesterase; LPS—lipopolysaccharide; TLR—Toll-like receptor; TTSS—type-III secretion system; Yops—*Yersinia* outer proteins; N6mA—N6-methyladenine; N4mC—N4-methylcytosine; 5mC—5-methylcytosine; LTTR—LysR-type transcriptional regulator.
